# Emotions and feelings in neuroscience education across career stages: a qualitative study with views from alumni, junior and senior academics

**DOI:** 10.1186/s12909-024-06546-0

**Published:** 2025-02-20

**Authors:** Stefano Sandrone

**Affiliations:** Department of Brain Sciences, Sir Michael Uren Hub, Imperial College London, 86 Wood Lane, London, W12 0BZ UK

**Keywords:** Emotions, Feelings, Neuroscience education, Neurology, Medical education, Stress, Anxiety, Self-efficacy, Identity, Mentoring

## Abstract

**Introduction:**

Emotions and feelings are crucial components of our lives. However, their role in medical education scholarship, including in neuroscience education, has been overlooked. Moreover, their impact has been understudied in academia, especially across career stages. We explored emotions and feelings in the context of neuroscience education and across different career stages.

**Methods:**

This work is based on a project exploring the value of learning in postgraduate education, drawing on data from nineteen semi-structured interviews with university alumni and academics. Eight alumni (six females and two males) and eleven academics from a UK-based STEM-intensive institution participated in the study. Alumni refers to former students who have completed the MSc in neuroscience at a STEM-intense institution within the last six years. Lecturers and Senior Lecturers in neuroscience have been labelled as *junior academics*, whereas Readers and Professors have been labelled *senior academics*.

**Results:**

The alumni recognised their master experience was intense and challenging, yet acknowledged that enjoyment and stress are two faces of the same coin. Many cited their peers as an inspiration to go beyond the limits but only one student mentioned gratitude, which was never mentioned by the academics. At least one alumnus and one junior academic mentioned trust and confidence, but not the senior academics. Anxiety and confidence (and lack of) dominated the interviews with junior academics, who used intense words when discussing the pleasures of science. Conversely, the senior academics dedicated few words to emotions and feelings, never talked about anxiety, trust or gratitude, and very briefly mentioned the reward linked to sharing their knowledge with the next generations.

**Discussion:**

This work epitomises the importance of exploring emotions and feelings in neuroscience education. Talking more openly about emotions and reducing the pervasive narration of ‘success stories’ might be directions to follow, along with stressing the importance of cultivating self-efficacy, trust and gratitude since the early stages. Meetings between students and supervisors can play a key role in developing self-trust among the students. More focus should be placed on supporting the transition process between postgraduate studies and the subsequent steps of the academic ladder.

**Supplementary Information:**

The online version contains supplementary material available at 10.1186/s12909-024-06546-0.

## Introduction

Emotions and feelings are crucial components of our life [47]. Mankind has reflected on them since ancient times [6], and several disciplines have examined these topics with various research approaches and from different perspectives [45]. But it is neuroscience, a relatively new discipline [13, 59], which ultimately shed light on cellular, molecular and system-related aspects of emotions and feelings [2, 20, 42]. From a neuroscientific perspective, emotions can be defined as ‘bioregulatory reactions that aim at promoting, directly or indirectly, the sort of physiological states that secure (…) survival regulated into the range that we (…) identify with well-being’ [12, 20]. Feelings are ‘the mental representation of the physiologic changes that occur during an emotion’ and include mapping ‘the changes that occur in the cognitive processing style, as well as the evocation of thoughts that are congruent with the feeling state’ [20, 21].

Still, the definitions of emotions and feelings go well beyond their physiological aspects, albeit universal. At least two additional levels can be added: they can be seen as a form of competence linked to skills and abilities but also framed within a ‘socio-cultural discourse which (…) directs our attention to emotion’s function in social exchanges and its role as a social, political and cultural mediator’ [44]. Emotions and feelings are key components of medical and scientific endeavours, from the excitement for a new idea to frustration [10, 33], from the empathy in the relationship between a patient and a physician [23, 76, 79] to pure joy for a treatment that works, through depression and anxiety of health care professionals [25]. Emotions and feelings pervade science and medicine as ‘practice, profession and social institution’ and ‘are elemental facets of scientists’ career evaluations and work life’ [24, 27, 49].

However, their role in medical education scholarship has often been less prominent than it could have been, and a decade ago it was labelled as an ‘ever-present absence’ [44]. Historically, they were neglected in studies on teaching and learning linked to identity development [38, 68]. The importance of these elements across the many levels of academic life is overlooked despite their undoubtedly relevant centrality in creating ‘social bonds, power-relationships and hierarchies’ and taking part in ‘processes of inclusion and exclusion from an academic career’ [7] (but see also [24] and [69]). Medical education can benefit by exploring the role of emotions in teaching and learning and by defining the impact of emotions on the training of healthcare professionals [43]. Also, the neuroscience postgraduate experience is under-studied despite the rising prominence of the discipline and the rapid global expansion of neuroscience courses [3, 62]. For the first time, here we investigate emotions and feelings in neuroscience education across different career stages by qualitatively analysing the views from alumni, junior and senior academics.

## Methods

This paper is based on a project exploring the value of learning in postgraduate education, drawing on data from nineteen semi-structured interviews with alumni and academics; the questions of the semi-structured interviews can be found in [58]. Eight alumni (six females and two males) and eleven academics (all males) from a UK-based STEM-intensive institution participated in this study. The label *alumni* indicates former students who completed the MSc in neuroscience at a STEM-intense institution within the last six years. Lecturers and Senior Lecturers in neuroscience are part of the *junior academics*, whereas *senior academics* include Readers and Professors. Each interview lasted less than an hour and was conducted via Teams. To avoid ‘engineering’ fake situations and triggering a social desirability bias [5], the emotions were not the explicit target of the interview questions but only discussed if and when spontaneously brought to the table by the participants. In these cases, specific follow-up questions were asked (i.e., ‘what do you mean?’, ‘how did you feel?’), as well as questions on unclear aspects [17, 64]. This project received ethical approval from the Education Ethics Review Process at Imperial College London (EERP2021-012) and informed consent to participate was obtained from all the participants. The study followed the guidelines of the British Educational Research Association [9]. Anonymised audio recordings were sent to Way With Words Ltd, a third-party transcription service, and transcriptions were analysed with thematic analysis [22]. The author embraced an inductive coding approach [48, 61]. The codes were not defined a priori but emerged from the thematic analysis [54]. This approach implied iterative readings and review of the transcriptions of the interviews. It allowed the author to embrace unexpected findings and capture the nuances, considering that little was known about emotions in postgraduate neuroscience. The author developed codes based on the concepts he came across, creating new codes as the analysis progressed. Saturation was reached.

## Results

**Fig. 1:**
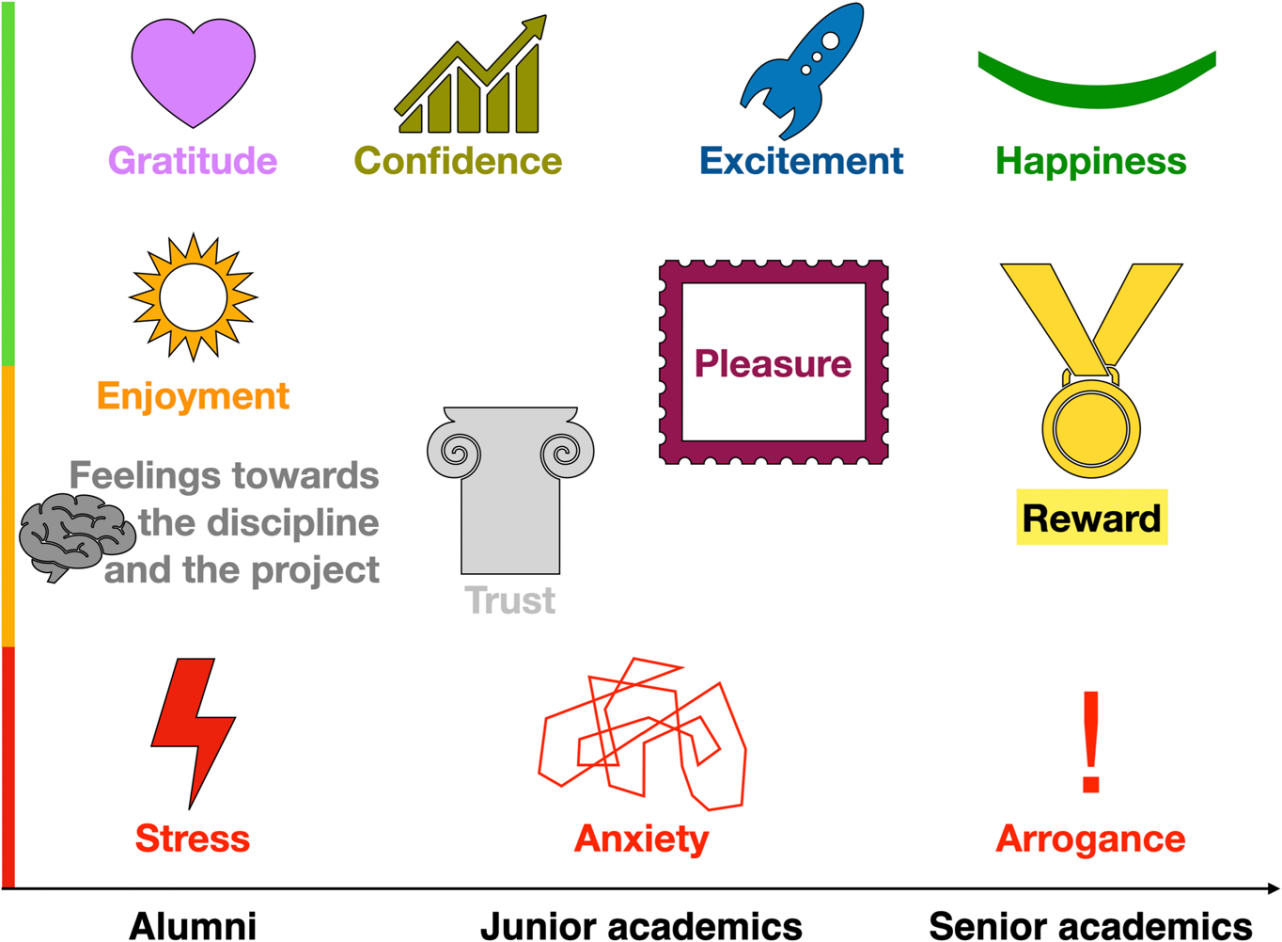
Summary of key emotions and feelings mentioned by the participants across different career stages

Several emotions and feelings were mentioned by the participants (Fig. 1). Five themes emerged: stress, anxiety, trust, confidence and enjoyment. However, not all the themes emerged at all the career stages (Table 1). The fact that themes seemed to vary by career stage is a finding in itself.

**Table 1:**
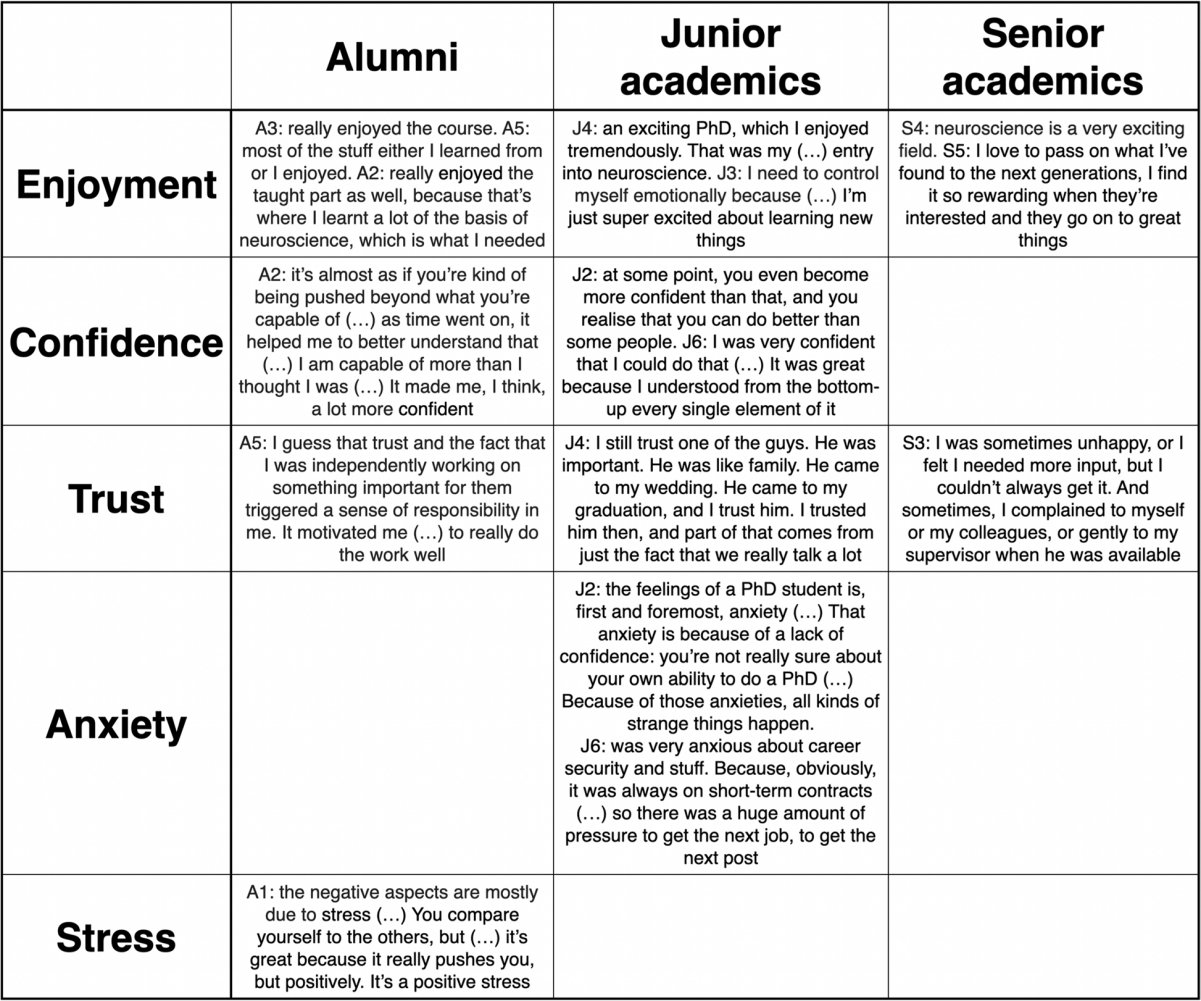
Illustrative quotes for the five themes that emerged during the thematic analysis: Stress, anxiety, trust, confidence and enjoyment. However, not all the themes emerged at all the career stages

### Alumni

#### Enjoyment and stress as two faces of the same coin

A3 was ‘really happy’ with master’s and ‘really enjoyed the course’. A6 recognised that the ‘course was quite intensive’. This was echoed by A2: ‘I’m sure everyone’s told you that it was a very challenging year, without a doubt, very intense’. The MSc had a ‘mainly positively’ impact on A1: ‘The negative aspects are mostly due to stress (…) You, obviously, have to sacrifice other things like more fun or seeing friends and family, but that’s the most negative effect (…). The positive ones: you become way more self-aware because (…) you’re within a group of very competitive and ambitious people. You also become a part of that mentality and mindset (…) what do I want to do, who do I want to become? You compare yourself to the others, but (…) it’s great because it really pushes you, but positively. It’s a positive stress’. A5 said that ‘most of the stuff either I learned from or I enjoyed’ despite the course being ‘quite intense’, and highlighted that ‘even if you’re not an academic, you’re going to have to present your work and talk about it all the time (…) That had a really big impact not just on me, but also on everyone I spoke to—they really enjoyed it. It was tough, it was scary at times, but they really enjoyed it afterwards’. On an enjoyment-related note, A2 ‘really enjoyed the taught part as well, because that’s where I learnt a lot of the basis of neuroscience, which is what I needed (…)’. However, the intensity somehow clashed with the possibility of fully appreciating what was learnt: ‘because it was so intense, it was very difficult to appreciate what you were doing along the way (…) you become like a robot almost’. Similarly, A5 ‘felt there was a lot of material to cover in a very short period of time and (…) you can’t delve into anything in particular, as the main aim becomes to just pass the exam (…) that can be quite negative’.

#### Feelings towards the research project

On ownership of the research project, A5 said that ‘the sense that I was getting from my supervisors was that they were interested in what I was doing (…) It was important not only for me as a student for my research project, but for them as well (…) Because the work I was doing was also their work, they were going to use my data, and potentially (…) could be published. I guess that trust and the fact that I was independently working on something important for them triggered a sense of responsibility in me. It motivated me (…) to really do the work well and to take it very seriously (…) I found something that I really loved, so that had a big impact (…) Even though it was a really challenging course for many people (…) having that sense of accomplishment almost made me realise my strength -that I could actually do it. Going through that, even though it was tough, it was really good. Like adversarial growth, almost (…) Sometimes people can be quite afraid to share (…) their thoughts for the future in terms of their research or what they want to do’. A7 highlighted the difference between the master’s project and the PhD project, mentioning a sense of ownership that was present also in the former, but more notable in the latter: ‘I did have the feeling that it was my project, but I knew that this part was just a small one. It’s a very short project (…), it’s a very limited amount of time, and there is a very limited amount of planning you do yourself. Obviously, there are elements that you do together with your PI or with the PhD student who supervises you. But it’s just because you don’t have that much time, it’s not the same as like in my PhD project (…) I felt more that it is a project of my own just because I had to go through the process myself, and feel like there is also more responsibility on me for this project’.

#### Feelings towards the discipline

Two alumni talked about their feelings towards the discipline. A4 was ‘just a little bit disappointed that the molecular neuroscience research isn’t of much interest to most students in the cohort’. Similarly, A8 ‘was really frustrated with molecular neuroscience because I felt like it has such a reductionist approach (…) Then going into the computational approaches, I think it was intimidating because you associate them with computer science and coding, and you don't know if you can do this (…) I was interested in lots of things (…) I was surrounded by people who (…) weren’t very enthusiastic about going to lectures on things that weren't directly linked to what they wanted to do (…) I think there were a lot of people who just wanted to be done with it and that was a little disheartening’.

#### Gratitude and confidence

Only one student, A2, mentioned gratitude and confidence: ‘there are a few things (…) that made me very grateful for the MSc itself (…) Also the peers are, obviously, intelligent people (…) That’s also something I was very grateful for, that I’ve got people around me that I can learn from rather than compete against (…) it’s almost as if you’re kind of being pushed beyond what you’re capable of (…) as time went on, it helped me to better understand that (…) I am capable of more than I thought I was (…) It made me, I think, a lot more confident’.

### Junior academics

#### Anxiety diminishes when confidence grows

Four out of six junior academics talked about emotions and feelings. J2 detailed the roles played by anxiety and confidence during the PhD, the postdoctoral years and as a junior academic: ‘The feelings of a PhD student is, first and foremost, anxiety. You’re just not sure where you’re going or whether this is leading to anything. So, that’s almost always with you at the beginning. At some point, you think, yes, this might be an okay thesis. Then you move to the next phase of: I think I’ve done it. So, that’s the feeling, but at the same time, you’re not completely confident. That anxiety is because of a lack of confidence: you’re not really sure about your own ability to do a PhD. But then, at some point, you realise, yes, maybe I can do an okay job. And then at some point, you even become more confident than that, and you realise that you can do better than some people. Eventually you decide, yes, I have something to contribute to this field, to this discipline. That confidence comes with experience and with being exposed to more and more people and different types of science and types of thinking, with trying to convince yourself that you can actually make something of your own that could be helpful to the progression of the field (…) Because of those anxieties, all kinds of strange things happen. The best you can do is to try to avoid them and move on from the issues that the field faces. But that’s sometimes inevitable: when your grant is being reviewed, all kinds of issues, which are not science-related, happen’. J6 added that, during their PhD, ‘I was left basically completely on my own to do it. So when I went and actually set up a lab from scratch, I was very confident that I could do that. (…) it was great because I understood from the bottom-up every single element of it. And I liked that level of control and the freedom to do what I wanted with it’. J6 was also ‘very anxious about career security and stuff. Because, obviously, it was always on short-term contracts (…) so there was a huge amount of pressure to get the next job, to get the next post. And then when I transitioned from one place to another, that was probably the worst for that because I did not actually want to be a postdoc anymore. I needed to get a real job. And that is a very difficult transition to make and to be confident that you're making the right decision (…) I think that was probably the biggest anxiety I had’.

#### Trust as a gateway to pleasure and excitement

J4 ‘had an exciting PhD, which I enjoyed tremendously. That was my (…) entry into neuroscience. A new field within neuroscience had just emerged, optogenetics, and it was fun to be at the edge of that (…) I’ve met some of the people involved (…) at a conference (…) I still trust one of the guys. He was important. He was like family. He came to my wedding. He came to my graduation, and I trust him. I trusted him then, and part of that comes from just the fact that we really talk a lot. Largely about stuff that had nothing to do with immediate work (…) The immediate work, for most of molecular biology (…) is boring, tedious, and being able to put it in a broader context was really fundamental’. J4 also reported a ‘gradual disillusion towards computational neuroscience (…) neuroscience community is an amorphic terminology. Within that, you have these small communities. People talk with each other because they publish on similar themes. It’s an interesting one, because this kind of small community, determines a lot about your feeling about the field. It could be a nasty small community, in that everybody’s trying to push down in the other. There could be a supportive community, and it’s exciting to meet other people at a conference and talk with them’. J2 remarked that ‘science is great: for example, when you get into a discussion at a meeting with another scientist, that’s pure pleasure. But the rest of it, the things that are more related to the issues connected to the funding, connected to the politics within the field, I have mixed feelings. I do not think they should be there for the most part’. J3 added: ‘I need to control myself emotionally because (…) I’m just super excited about learning new things. I've always felt like I didn’t want to be an academic who only cares about one thing (…) my inner self will naturally push me to spread myself across a lot of things. But I've learned (…) that I need to prioritise’.

### Senior academics

#### (Un)happiness and reward

Four out of five senior academics talked about emotions and feelings. S3 reflected on pre-tenured life, starting from a recount of the PhD: ‘I had to find solutions to many problems (…) I first tried to find solutions myself, as opposed to going and asking my supervisor. This sink-or-swim approach was extremely valuable to me. I am not sure I realised this fully at the time, but I do realise fully now. At that time, I was sometimes unhappy, or I felt I needed more input, but I couldn’t always get it. And sometimes, I complained to myself or my colleagues, or gently to my supervisor when he was available (…) In hindsight, I think that it was the best possible scenario for learning and for developing independence (…) If it were so extreme as to not have had a supervisor and not to have received any input, then it wouldn’t have worked, and I wouldn’t be making this judgment today. (…) In hindsight: A, I have been successful, even with minimal supervision, and, B, the minimal supervision has given me great freedom and great responsibility (…) Yet the postgrad study is a sprint at the beginning of a marathon’. S5 was more forward-looking: ‘I love to pass on what I’ve found to the next generations, I find it so rewarding when they’re interested and they go on to great things. I’ve had PhD students who are faculty, who’ve gone into industry, they’ve maintained that *joie de vivre* and they love what they do. And if I’ve helped in any way, I find that rewarding’.

#### Arrogance (and lack of arrogance)

S1 and S4 mentioned arrogance and lack of arrogance in the world of neuroscience academia. S1 recounted a story about a leader in the field, ‘a professor in the US (…) who has done lots of original work (…) and she’s an absolute leader in this field (…) is the only person I have seen who has more than 20 or 30 publications in NEJM. Then, I compare this with other academics who get a couple of NEJM papers and they’re on the top of the world and just become so arrogant. She was just so down to earth, such a simple person, but one of the most widely respected neurologists around the globe’. S4 highlighted that neuroscience is a very exciting field and said that ‘both my PhD supervisor and the more recent mentor are, what’s the best way to put it, on paper extremely successful but neither of them is particularly arrogant (…) I think many people, if they’re honest about it, would say that academics do tend to have, like everybody, egos that can be damaged and bigger egos I guess. People often do academic work because they want to be, in inverted commas, the best at what they do, or to make a massive discovery, or to change the world. They don’t do things silently, and silent academics won’t get very far. So I think by its very nature academia does involve some self-aggrandisement, and so it’s important not to get carried away with that (…) the reasons for this in academia are the competition, the nature of funding (…), the scarcity of positions’.

## Discussion

Not all the participants spontaneously talked about emotions and feelings. When they opened up about these topics, positive and negative aspects were covered, albeit in different proportions and in different ways.

The alumni recognised that their master's experience was intense and challenging, yet acknowledged and accepted that enjoyment and stress are two faces of the same coin. Many cited their peers as a stimulus to go beyond the limits and expectations. Only one student mentioned gratitude, which was never mentioned by the academics. At least one student and one junior academic mentioned trust and confidence (from the two perspectives of being supervised, for the former, and both as supervised and supervisor, for the latter), but not the senior academics. Anxiety and confidence (and lack of) dominated the interviews with junior academics, who generally used more intense words when discussing the pleasures of science. Conversely, the senior academics dedicated few words to emotions: they never cited anxiety, trust or gratitude. Still, they talked about arrogance experienced (or not experienced) and mentioned the reward of sharing their knowledge with the next generations *en passant*. A sort of ‘temporal detachment’ might be one of the reasons behind this: while the students talk about their own learning experience just days or months after completing their master's, senior academics were interviewed years after reaching the top of the academic ladder.

Stress and anxiety had a leading role in many interviews, especially among the alumni and junior academics (the former mentioned by the alumni, the latter by junior academics, mainly when talking about their doctoral and postdoctoral studies). While this was somehow expected, the lack of specific references to anxiety on the alumni side is surprising. The sacrifices of students often go ‘unnoticed and unspoken’ [55], and a ‘culture of self-sacrifice’ has structurally become part of the hidden curriculum in many universities [51]. This can lead to stressful situations that make the learning experience less enjoyable, as some students highlighted. Although multiple definitions of stress exist [15], a negative association between quality of living and stress has been overall widely reported among university students [1, 53], a population where anxiety (and depression) are, unfortunately, highly prevalent [63]. The PhD time itself was defined as ‘turbulent’ in a *Nature*’s survey conducted with 6,300 graduate students: 36% of respondents reportedly sought help for anxiety or depression [34, 75]. In a meta-analysis focusing on the prevalence of clinically significant symptoms across 15,626 students, the estimated proportion of learners with anxiety was 0.17 [63]. Reportedly, females scored higher than males on anxiety and stress, and first-year students were more anxious than their third-year colleagues [8]. The COVID-19 pandemic has exacerbated this situation: students display significantly higher anxiety rates after the pandemic compared to pre-pandemic levels [8].

While the sources of emotional strain and concerns can be many, a recurrent reason is the uncertainty related to job prospects, followed by the work-life balance ([75]; Je et al., 2022). Academic and financial stressors were also mentioned by 2,582 PhD students at five U.S. public research universities [37]. Something can and should be done. Mentoring can mediate the correlation between research self-efficacy and depression/anxiety, as postulated by a cross-sectional study with 325 PhD candidates in biomedicine [41]. Improving the sense of belonging might reduce students’ odds of anxiety and depression symptoms [37] and inserting self-care modules can be a step forward [51]. Another avenue to follow might be to increase self-confidence -not only before access to university but also during the bachelor's and master’s years. Individuals might decide not to pursue a STEM-related career or study path because of the difficulties they encounter and the associated negative emotional states, which are often linked to a self-reinforcing low sense of confidence and a lack of persistence [35, 67]. The supervisor's curiosity during supervision meetings can foster confidence and self-trust in students, as shown by a recent qualitative study where supervisory meetings were video-recorded and analysed [39]. These meetings can be instrumental towards developing self-trust among the students; also, within an academic setting, care can be seen as a relational structure linked to practices of curiosity and the act of sharing tacit knowledge [39]. Beyond self-trust, trust is the missing component in dysfunctional teams and a key component in successful team collaborations [26, 52, 56], as also highlighted by some interviewees. Learners emphasised the importance of supervisor flexibility to match their learning needs [65, 66]. Future reports can analyse how a post-pandemic world increasingly based on hybrid and asynchronous work [14] can further exploit the flexibility the learners want without compromising the quality of academic supervision.

On a supervisory dynamics-related note, little space was dedicated to gratitude by the participants. But it is gratitude, along with forgiveness and acceptance, that predicts resilience among university students, with gratitude being the highest predictive value [30, 78]. In undergraduate courses, gratitude was linked to high levels of student satisfaction, and gratitude levels peak during the final year of studies [19, 78], but we did not replicate this as only one alumnus out of eight mentioned gratitude. Gratitude can be nurtured, as shown by a qualitative case study investigating the impact of gratitude as an intervention over a temporal window of six weeks: the participants, eight PhD students and two supervisors, noted a positive impact on communication, social and emotional well-being and research process [29]. Feelings of gratitude are driven by helping behaviours, care, perceived effort and environment [19, 77], and future studies can analyse each of these elements across different contexts in neuroscience education and weigh their importance across career stages. More emphasis should be placed on supporting the transition process between undergraduate and postgraduate and beyond [28] and on showcasing the perks that a career in neuroscience can offer. We need to cultivate foundations of self-trust, trust and gratitude in academia, starting from the undergraduate and master’s levels.

Moving from the PhD to the postdoc, it is known that high levels of competition characterise the postdoc life. Some participants mentioned the lack of support from the community despite receiving socio-emotional and informational support, which is often fundamental for postdocs and academics in STEM [46, 73]. Different forms of support might mediate post-PhD researchers’ (dis)engagement in the early stages of their careers [18, 73]. More generally, few works explored ‘how emotionally powerful work experiences influence post-PhD researchers’ identity development’: work experiences are significant when they help scientists gauge if their self-representation as researchers is coherent and whether a further research career is viable [32, 68, 74]. Positive experiences sustained their motivation and made them feel they were consolidating their identities, whereas negative experiences challenged their identity and sense of belonging towards academia. While positive feelings persisted over time, negative feelings had greater saliency but faded away or evolved through self-reflection [68, 71] (but see also [57] and [72]). Neuroscience academics should reduce the narration of ‘success stories’ [11, 40] to emphasise, instead, how things can be learnt from negative experiences. Medical education studies can analyse personal and career-related trajectories with current and former academics to see if the ‘four trajectories of academic identity development (one of stable academic identity and three of lost academic identity) and four narratives of attrition (disillusionment, a search for new purpose, refusal to sacrifice personal life and academic inadequacy)’ [16] can be replicated in neuroscience education.

In our study, the senior academics did not extensively mention the reward linked to mentoring the next generations of scientists, which was indicated as one of the most positive aspects of academia, along with academic freedom and flexible schedules in a survey with 244 winners of the National Science Foundation Graduate Research Fellowships; in contrast, pressures for funding, publication and tenure were the negative ones [58] and were mentioned in our work too. Most scientists, also in our study, feel satisfied when they publish, as they feel they contributed to the overarching scientific endeavour, and satisfaction lasts longer when age increases [31, 36]. However, publishing in biological sciences positively stimulates older, experienced scientists but may still stress young researchers [4, 31]. Moreover, the job satisfaction of scientists is not always equally perceived by people born and working in different countries, which makes the entire situation even more complex. For instance, despite higher research productivity, foreign-born scientists are less satisfied than native-born peers [50, 60]. Our work did not delve deeper into such cultural elements, which can be the focus of future studies, along with tensions and nuances of the emotions surrounding the academic journey.

This work has limitations and strengths. Although independent prior coding by two researchers is usually recommended, this is a single-authored, single-centred study, but one where follow-up questions were asked to clarify ambiguous aspects. The sample size of this educational study is adequate to achieve data saturation and an in-depth description of the identified themes, which are positive signs. This analysis covers three career stages, which is another strength. The qualitative methods offer a rich opportunity to explore this [70]. Also, emotions were not the explicit target of the interview questions to avoid ‘engineering’ fake situations and triggering a social desirability bias, but only discussed if spontaneously brought to the table by the participants. Moreover, this work provides a record of the answers given by each interviewee (Supplementary file).

This exploratory and pioneering work epitomises the importance of investigating emotions and feelings in a relatively young discipline like neuroscience, which, more than any other discipline, has contributed to the definition of emotions and feelings in neurobiological terms. Beyond the specific research avenues discussed above, emotions and feelings should be given more space in the scholarly universe of medical education *tout court* and can become crucial assets to sustain and boost neuroscientists’ careers at different stages.

## Supplementary Information

Supplementary Material 1. Record of the answers provided by the interviewees when mentioning or discussing emotions and feelings (Supplementary file).

## Data Availability

The dataset analysed in this study is available and can be found in the Supplementary File.
