# Supplementary material for: Emotions and feelings in neuroscience education across career stages: a qualitative study with views from alumni, junior and senior academics
Source: BMC Med Educ. 2025 Feb 20;25:277. doi: 10.1186/s12909-024-06546-0 (PMC11843773; doi:10.1186/s12909-024-06546-0)
Supplement: Supplementary file 1 — Supplementary Material 1. Record of the answers provided by the interviewees when mentioning or discussing emotions and feelings (Supplementary file). [file 12909_2024_6546_MOESM1_ESM.docx]

**Alumni**

**A1:** Mainly positive (…) The negative aspects are mostly due to stress (...) You, obviously, have to sacrifice other things like more fun or seeing friends and family, but that’s the most negative effect (…). The positive ones: you become way more self-aware because (…) you’re within a group of very competitive and ambitious people. You also become a part of that mentality and mindset (…) What do I want to do, who do I want to become? You compare yourself to the others, but (…) it’s great because it really pushes you, but positively. It’s a positive stress.

**A2:** I’m sure everyone’s told you that it was a very challenging year, without a doubt, very intense (…) There are a few things (…) that made me very grateful for the MSc itself (…) Also the peers are, obviously, intelligent people (…) That’s also something I was very grateful for, that I’ve got people around me that I can learn from rather than compete against (…) it’s almost as if you’re kind of being pushed beyond what you’re capable of (…) as time went on, it helped me to better understand that (…) I am capable of more than I thought I was (…) It made me, I think, a lot more confident (…) I really enjoyed the taught part as well, because that’s where I learnt a lot of the basis of neuroscience, which is what I needed (…) because it was so intense, it was very difficult to appreciate what you were doing along the way (…) because you become like a robot almost, you’re thinking that I need to do this, do this, do this, because I’ve got an exam to do (…) I think everyone felt that in a way (…) we have to get it done, so we have to do this rather than actually appreciate what we’re learning.

**A3:** Really happy with Master’s, really enjoyed the course.

**A4:** I’m just a little bit disappointed that the molecular neuroscience research isn’t of much interest to most students in the cohort.

**A5:** The sense that I was getting from my supervisors was that they were interested in what I was doing (…) It was important not only for me as a student for my research project, but for them as well (…) Because the work I was doing was also their work, they were going to use my data, and potentially (…) could be published. I guess that trust and the fact that I was independently working on something important for them triggered a sense of responsibility in me. It motivated me (…) to really do the work well and to take it very seriously (…) I found something that I really loved, so that had a big impact (…) Even though it was a really challenging course for many people (…) having that sense of accomplishment almost made me realise my strength -that I could actually do it. Going through that, even though it was tough, it was really good. Like adversarial growth, almost (…) Sometimes people can be quite afraid to share (…) their thoughts for the future in terms of their research or what they want to do (…) Most of the stuff either I learned from or I enjoyed (…) it was quite intense, and maybe having that pace slower, so that people are more able to dig into certain topics… Because I felt there was a lot of material to cover in a very short period of time and (…) you can’t delve into anything in particular, as the main aim becomes to just pass the exam (…) that can be quite negative, that can have a not very good impact on education, in general (…) Even if you’re not an academic, you’re going to have to present your work and talk about it all the time (…) That had a really big impact not just on me, but also on everyone I spoke to—they really enjoyed it. It was tough, it was scary at times, but they really enjoyed it afterwards.

**A6:** The course was quite intensive.

**A7:** I did have the feeling that it was my project, but I knew that this part was just a small one. It’s a very short project (…), it’s a very limited amount of time, and there is a very limited amount of planning you do yourself. Obviously, there are elements that you can do together with your PI or with the PhD student who supervises you. But it’s just because you don’t have that much time, it’s not the same as like in my PhD project (…) I felt more that it is a project of my own just because I had to go through the process myself, and feel like there is also more responsibility on me for this project.

**A8:** I was really frustrated with molecular neuroscience because I felt like it has such a reductionist approach (…) Then going into the computational approaches, I think it was intimidating because you associate them with computer science and coding, and you don't know if you can do this (…) I was interested in lots of things (…) I think that was just what was intimidating because I was surrounded by people who (…) weren’t very enthusiastic about going to lectures on things that weren't directly linked to what they wanted to do (…) I think there were a lot of people who just wanted to be done with it and that was a little disheartening.

**Junior Academics**

**J2:** The feelings of a PhD student is, first and foremost, anxiety. You’re just not sure where you’re going or whether this is leading to anything. So, that’s almost always with you at the beginning. At some point, you think, yes, this might be an okay thesis. Then you move to the next phase of: I think I’ve done it. So, that’s the feeling, but at the same time, you’re not completely confident. That anxiety is because of a lack of confidence: you’re not really sure about your own ability to do a PhD. But then, at some point, you realise, yes, maybe I can do an okay job. And then at some point, you even become more confident than that, and you realise that you can do better than some people. Eventually you decide, yes, I have something to contribute to this field, to this discipline. That confidence comes with experience and with being exposed to more and more people and different types of science and types of thinking, with trying to convince yourself that you can actually make something of your own that could be helpful to the progression of the field (…) Because of those anxieties, all kinds of strange things happen. The best you can do is to try to avoid them and move on from the issues that the field faces. But that’s sometimes inevitable: when your grant is being reviewed, all kinds of issues, which are not science-related, happen. Science is great: for example, when you get into a discussion at a meeting with another scientist, that’s pure pleasure. But as for the rest, the things that are more related to the issues connected to funding, connected to politics within the field, I have mixed feelings. I do not think they should be there for the most part.

**J3:** I need to control myself emotionally because (…) I’m just super excited about learning new things. I've always felt like I didn’t want to be an academic who only cares about one thing (…) my inner self will naturally push me to spread myself across a lot of things. But I've learned over time that I need to prioritise.

**J4:** Gradual disillusion towards computational neuroscience (…) I’ve met some of the people involved (…) at a conference (…) I still trust one of the guys. He was important. He was like family. He came to my wedding. He came to my graduation, and I trust him. I trusted him then, and part of that comes from just the fact that we really talk a lot. Largely about stuff that had nothing to do with immediate work (…) The immediate work for most of molecular biology (…) is boring, tedious, and being able to put it in a broader context was really fundamental (…) I had an exciting PhD, which I enjoyed tremendously. That was my (…) entry into neuroscience. A new field within neuroscience had just emerged, optogenetics, and it was fun to be at the edge of that (…) I think that neuroscience community is an amorphic terminology. Within that, you have these small communities. People talk with each other because they publish on similar themes. It’s an interesting one, because this kind of small community determines a lot about your feeling about the field. It could be a nasty small community, in that everybody’s trying to push others down. There could be a supportive community, and it’s exciting to meet other people at a conference and talk with them.

[PhD to postdoc]: it wasn’t a shock as a bad shock, it was all super excited. I think, like many scientists, I’m driven by curiosity. That was so much to discover and then to learn. And I just embraced it. So, I didn’t see it as a bad experience at all. I think it was a very good experience to do that, it was just very, very different. I think the best way is, and it is what’s happened to me, basically just learning by doing.

**J6:** And I was left basically completely on my own to do it. So when I went and actually set up a lab from scratch, I was very confident that I could do that. (…) It was great because I understood from the bottom-up every single element of it. And I liked that level of control and the freedom to do what I wanted with it (…) I would say there was never any anxiety relating to that, but I was very anxious about career security and stuff. Because, obviously, it was always on short-term contracts (…) so there was a huge amount of pressure to get the next job, to get the next post. And then when I transitioned from one place to another, that was probably the worst for that because I did not actually want to be a postdoc anymore. I needed to get a real job. And that is a very difficult transition to make and to be confident that you're making the right decision (…) I think that was probably the biggest anxiety I had.

**Senior academics**

**S1:** A professor in the US (…) She is a person who has done lots of original work (…) and she’s an absolute leader in this field (…) she is the only person I have seen who has more than 20 or 30 publications in NEJM. Then, I compare this with other academics who get a couple of NEJM papers and they’re on the top of the world and just become so arrogant. She was just so down to earth, such a simple person, but one of the most widely respected neurologists around the globe.

**S3:** I had to find solutions to many problems (…) I first tried to find solutions myself, as opposed to going and asking my supervisor. This sink-or-swim approach was extremely valuable to me. I am not sure I realised this fully at the time, but I do realise fully now. At that time, I was sometimes unhappy, or I felt I needed more input, but I couldn’t always get it. And sometimes, I complained to myself or to my colleagues, or gently to my supervisor when he was available (…) In hindsight, I think that it was the best possible scenario for learning and for developing independence (…) If it were so extreme as to not have had a supervisor and not to have received any input, then it wouldn’t have worked, and I wouldn’t be making this judgment today. (…) In hindsight: A, I have been successful, even with minimal supervision, and, B, the minimal supervision has given me great freedom and great responsibility. And by taking that responsibility, I’ve been able to learn more, to develop a degree of independence, which I think would have been much slower to develop in more tightly supervised circumstances. (…) It’s always good to try and keep a balance (…) Yet the postgrad study is a sprint at the beginning of a marathon.

**S4:** Both my PhD supervisor and the more recent mentor are, what’s the best way to put it, on paper extremely successful but neither of them is particularly arrogant (…) I think many people, if they’re honest about it, would say that academics do tend to have, like everybody, egos that can be damaged and bigger egos I guess. People often do academic work because they want to be, in inverted commas, the best at what they do, or to make a massive discovery, or to change the world. They don’t do things silently, and silent academics won’t get very far. So I think by its very nature academia does involve some self-aggrandisement, and so it’s important not to get carried away with that (…) the reasons for this in academia are the competition, the nature of funding (there are not too many), the scarcity of positions. Why? Because there are many competitive sectors in the world and everybody clearly aims to do something in the best way possible. But success (…) in academia is dependent on those things to some extent. So to be successful in academia everybody has to know you are the expert and acknowledge it, otherwise it’s more difficult to get the next grant, it’s more difficult to get the next manuscript in (…) People (…) will always prefer a *Nature* paper than one in a much smaller less impactful journal (…) neuroscience is a very exciting field.

**S5:** I love to pass on what I’ve found to the next generations, I find it so rewarding when they’re interested and they go on to great things. I’ve had PhD students who are faculty, who’ve gone into industry, they’ve maintained that *joie de vivre* and they love what they do. And if I’ve helped in any way, I find that rewarding.
